# Processing of Chinese Base-Generated-Topic Sentences by L1-Korean Speakers: An Eye-Tracking Study

**DOI:** 10.3390/brainsci12111573

**Published:** 2022-11-18

**Authors:** Kaiyan Song, Hui Chang, Yuxia Wang

**Affiliations:** School of Foreign Languages, Shanghai Jiao Tong University, Shanghai 200240, China

**Keywords:** Chinese base-generated-topic sentences, eye-tracking, shallow structure hypothesis

## Abstract

According to the shallow structure hypothesis (SSH), adult L2 learners rely more on lexical-semantic and pragmatic information but less so on syntactic information in online language processing, ending up with shallower syntactic representation. To test the SSH, we conducted an eye-tracking experiment on L1-Korean L2-Chinese learners with native Chinese speakers as the baseline, investigating their processing of Chinese base-generated-topic sentences (BGT). The results show that both the intermediate and advanced Korean learners of Chinese are sensitive to and can make use of syntactic information, but only the advanced learners are sensitive to the semantic constraint when processing Chinese BGT sentences, providing evidence against the SSH.

## 1. Introduction

For second language learners, online processing and comprehending the target input is important. According to the shallow structure hypothesis (SSH) [1,2], there is a fundamental difference in sentence processing between native speakers and non-native adult L2 speakers. The SSH claims that adult L2 learners fail to make a full use of syntactic information and rely more on lexical-semantic and pragmatic information in sentence processing. Thus, the SSH suggests that compared with native speakers, adult L2 learners’ syntactic representations are relatively shallower with less syntactic detail. This suggests that L2 learners’ sentence processing is qualitatively different from native speakers. L2 learners seldom utilize structure-based processing strategies in solving ambiguities in L2 processing.

Many empirical studies in support of the SSH mainly come from the processing of relative-clause and filler-gap dependencies, revealing sensitivity to nongrammatical information and reduced dependency on (morpho-)syntactic information during L2 sentence processing [3,4]. For example, Dinçtopal-Deniz [5] investigated the processing of relative clause attachment ambiguities by advanced L2 English learners with Turkish as their native language based on a self-paced reading task and an offline pen-and-paper questionnaire. The results showed that the attachment preference of the L2 learners was not similar to either English native speakers or monolingual Turkish speakers, and their attachment preferences were more guided by lexical-semantic information instead of syntactic information, providing evidence for the SSH. Besides, other findings concerning relative clause ambiguities [6,7] also demonstrated sensitivities to lexical-semantic cues but no clear syntactic disambiguation preference without semantic information. Moreover, in terms of filler-gap dependencies, Marinis et al. [8] used a self-paced reading task to investigate online processing of long-distance *wh*-dependencies from four groups of L2 learners of English with different L1 backgrounds (Chinese, Japanese, German and Greek). Different from the native speakers who showed sensitivity to syntactic gaps in processing, the L2 learners related the fronted *wh*-phrase to its lexical subcategorizer directly, irrespective of the subjacency constraint. This result suggested the L2 learners’ underuse of syntactic information, providing evidence for the SSH.

Nevertheless, the SSH has also been challenged by some studies. Omaki and Schulz [9], using a self-paced reading task, compared the differences between L1-Spanish L2-English learners and English native speakers in processing the relative clause island constraint of constructing filler-gap dependencies. Both the native speakers and L2 learners presented evidence of the application for the relative clause island constraint, providing evidence against the SSH. In addition, Pliatsikas and Marinis [10] investigated L2 learner’s processing of *wh*-dependencies with a self-paced reading task and found that L2 learners with naturalistic exposure provide evidence of native-like processing. They argued that linguistic immersion may contribute to the abstract syntactic processing in L2.

It is seen that the above-mentioned studies either for or against the SSH mostly involve relative clause and *wh*-dependencies, both of which contain syntactic gaps inside the sentence. In other words, the sentence types used to attest for the SSH were not rich enough and basically confined to these structures with syntactic gap. Moreover, in English relative clause and *wh*-dependencies, the fronted *wh*-word would leak out the status of potential filler due to the *wh*- marking, hence it gives the parser a hint of the incoming gap.

A typical type of sentence that contains no syntactic gap is Chinese base-generated-topic (BGT) sentences. Chinese is well-known as a topic-prominent language mainly because it allows BGT sentences, often referred to as Chinese-style topic sentences [11,12]. In Chinese BGT sentences, the topic is generated from its original position, instead of from movement. Being independent of the verb, the topic is not an argument of the sentence. Therefore, the topic has no syntactic relations with any constituents in the remaining part of the sentence, that is, there is no syntactic gap. In contrast, English does not allow any BGT sentence, and the English topic sentences are even rare occurring in colloquial and oral conversations. For example, the sentence in (1) is an English topic sentence, where the topic NP “banana” is originally the object of the verb “like”. After topicalization, which means to move from the object position to the sentence initial position, it leaves a syntactic gap at the object position.

(1) Bananas, I like most.

Some literature [13,14,15] held different views and analysis for syntactic movement. For example, Rizzi [15] proposed A’ chain analysis, which included two unique positions, the s-selection position (normally the θ-position) and criterial position. These positions would lead to a ban on movement to θ-position and movement from a criterial to a criterial position. However, this view was questioned and opposed by considerable literature, such as in [16,17,18]. 

According to the split CP hypothesis [19,20], a CP can be split into a force phrase, a topic phrase, a focus phrase, and so on. Likewise, the Chinese topic sentence was also regarded as a topic phrase (TopP) by many linguists [21,22,23]. Specifically, in Chinese the TopP is the maximal projection of the head occupied by the topic marker which may be null. The topic locates in the specifier position, while the comment, normally an IP or TP serves as the complement of TopP [24]

In the Chinese BGT sentence of example (2) [25], the topic *shuiguo* “fruit” is generated from its original position and it’s not an argument of the verb, *aichi* “love to eat”, which takes *xiangjiao* “banana” as its argument rather than the topic NP. As a result, the topic *shuiguo* “fruit” bears no syntactic relation with any other constituent in rest sentence, forming a “gapless structure”. 

(2) *Shuiguo wo zui ai chi xiangjiao.*

Fruit I most love eat banana.

‘As for fruits, I like bananas most.’

Semantically, the topic *shuiguo* “fruit” associates with the NP *xiangjiao* “banana” in the following comment in a superordinate-hyponymy relation. Other relations like hyponymy-superordinate relation shown in (3) [25] or sisterhood relation shown in (4) [25] make Chinese BGT sentences semantically unacceptable. This semantic constraint has been explained by the topic licensing condition [26,27], which states that a topic is licensed if there is a variable in the comment and the set generated by this variable produces a non-empty set when intersecting with the set represented by the topic. 

(3) *xiangjiao wo zui ai chi shuiguo.

Banana I most love eat fruit.

‘*Bananas, I like to eat fruit the most.’

(4) *Pingguo wo zui ai chi xiangjiao.

Apple I most love eat banana.

‘*Apples, I like to eat banana the most.’

Because of these unique characteristics, Chinese BGT sentences have drawn much attention. In the field of L1 research, Xu and Langendoen [28] systematically analyzed the form and main features of Chinese BGT sentence. Besides, Shi [21] specified several subtypes of Chinese BGT sentences, and discussed their properties. Moreover, Pan and Hu [26,27] further investigated the syntactic and semantic relations between the topic and its related NP in the Chinese BGT sentences. In terms of L2 research, many studies explored the acquisition of Chinese BGT sentences with off-line methods. For example, Yuan [29] investigated the acquisition of Chinese base-generated topics by adult English native speakers with an acceptability judgment test. The study found that although English speakers were exposed to positive evidence of base-generated topics when learning Chinese, it was rather difficult for them to acquire Chinese base-generated topics. Specifically, the study found that Chinese BGT sentences were typically deemed unacceptable by learners ranging from the beginner level to the intermediate level, and there was noticeable improvement in acceptance when learners reached an advanced level. In contrast, using a grammaticality judgment task, Liu [30] found that L1-English L2-Chinese learners with lower proficiency can acquire Chinese BGT sentences at an early stage. Including leaners other than L1 English speakers, Cao et al. [31] investigated the L2 acquisition of Chinese topic sentences by English, Korean, and Japanese speakers with an acceptability judgment task containing moved and base-generated -topic sentences. The results showed that Korean and Japanese speakers tended to find Chinese topic sentences more acceptable than English speakers, suggesting a transfer effect. Further, the moved Chinese topic sentences were generally easier to acquire than the Chinese BGT sentences. Different acquisition patterns between moved and based-generated topic sentences were also found in Hu et al. [32], which explored Japanese and English speakers’ acquisition of Chinese topic sentences with the HSK Dynamic Composition Corpus. The results showed that English and Japanese speakers could produce moved Chinese topic sentences but very few Chinese BGT sentences, suggesting that Chinese BGT sentences are difficult for them at least in the production task. It remains to be further investigated by online methods.

In sum, research in the past has largely supported that Chinese BGT sentences are generally challenging for L2 learners and the acquisition of Chinese BGT sentences might only occur with learners at the advanced level. Although these studies included learners with different proficiency and different native languages, there are a few issues that are under-researched. One is the consideration of semantic constraint and how this interacts with the acquisition of Chinese BGT sentences. The other consideration is the method. Tasks used in previous studies were offline, and therefore did not allow us to investigate the processing of Chinese BGT sentences in real-time. Online tasks would enable us to better observe the process that participants were engaged in when going through different elements of a Chinese BGT sentence, including the topic, the subject, and the NP in the comment relating to the topic, allowing us to find out which part triggers difficulties.

Two studies have so far investigated the processing of Chinese BGT sentences and addressed semantic constraints in Chinese BGT sentences. Yuan [25] conducted a self-paced reading experiment with advanced L1-English L2-Chinese speakers. The experiment included four sentence types, (a) acceptable Chinese BGT sentences where the topic and its related NP in the comment form a superordinate-hyponym relation, as shown in (2) above, (b) unacceptable sentences where the topic and its related NP in the comment form a hyponym-superordinate relation which violates the semantic constraint, as shown in (3) above, (c) unacceptable sentences where the topic and its related NP in the comment bears a sisterhood relation, violating the semantic constraint, as shown in (4) above, and d) acceptable non-BGT sentences beginning with an adverbial, which usually appears before the subject. The results revealed that L1-English speakers were sensitive to the semantic constraint and were able to identify the topic and subject in a native-like way in processing Chinese BGT sentences. Yuan [25], however, only recruited advanced L2 learners, but intermediate L2 learners still remain to be studied. Additionally, Zeng et al. [33] investigated intermediate and advanced L1-Vietnamese L2-Chinese speakers’ processing of Chinese BGT sentences with a self-paced reading task. The results showed that both intermediate and advanced L1-Vietnamese L2-Chinese speakers were sensitive to the semantic constraints and performed in a native-like way in identifying the topic and the subject.

Moreover, Yuan [25] and Zeng et al. [33] adopted a self-paced reading task, which is an unnatural way of reading since it breaks the whole sentence into segments and does not allow for regressions. As a result, the self-paced reading task only yields a single reading measure of each region in the sentence and it’s not the best way to investigate the process of restructuring and reanalysis that may happen during L2 processing [34,35,36]. 

One promising research methodology for capturing the whole picture of on-line sentence processing is the eye-tracking technique, which has multiple benefits. For one, it ensures a more natural way of reading instead of breaking the sentence into segments in self-paced reading tasks. For another, it presents a multifaceted trace of the reader’s processing with different reading measures, providing a better way to observe the regression and reanalysis [37,38]. To our knowledge, eye-tracking studies of Chinese topic sentences are rare, since previous studies of L2 Chinese sentences processing with eye-tracking mostly involve the Chinese relative clause and scope.

Another contribution was that we focused on L1-Korean speakers in our investigation. Different from English, which is typologically a subject-prominent language [12], or Vietnamese which is a topic-prominent language [39], Korean is a both topic-prominent and subject-prominent language with abundant morphological devices [12]. Taking into consideration of the characteristics of learners’ first language is critical as L1 serves as a filter for different components of L2 system [40]. It has been widely reported that L2 learners are susceptible to L1-spefific processing strategies in processing L2 input [37,41,42,43,44]. For example, Hopp [44] proposed that one of the major differences between L1 and L2 sentence processing is that L2 learners use L1-based parsing strategies in processing L2 sentences. By including L1-Korean speakers whose L1-based parsing strategies are different from the ones of speakers of English or Vietnamese, we aimed to explore whether the native-likeness can be achieved by L1 Korean (a topic-prominent and subject prominent language) speakers in L2 Chinese (a topic-prominent language) sentence processing.

Specifically, the current study aimed to investigate how L1-Korean speakers process Chinese BGT sentences, particularly how they process the topic and subject, and whether they are sensitive to the syntactic information and semantic constraint underlying the Chinese BGT sentences.

## 2. Base-Generated-Topic Sentences and Parsing Strategies in Chinese and Korean

Korean is a language that is both topic-prominent and subject-prominent [12]. Korean topic sentences have some similarities to Chinese. For instance, the topic generally appears in the sentence-initial position and it is what is being talked about in the comment. More importantly, like Chinese, Korean allows both moved and base-generated-topic sentences. For example, in (5) the topic ku yenghwan-un “this movie” is a moved topic from the object position of *poassta* “seen”. In contrast, in (6), a Korean version of (2) above, the topic *kwail-un* “fruit” is base-generated as it is generated in the original position. 

(5) Ku yenghwan-*un* nay-*ka* imi poassta.

This movie I have…before seen.

‘This movie I have seen.’

(6) Kwail-*un* nay-*ka* panana-*lul* kacang cohahanta. 

Fruit I banana most love eat.

‘As for fruits, I like bananas most.’

Similar to Chinese, base-generated topics in Korean are supposed to have certain semantic relations with the NP in the comment as well [45] (Kiss, 1995). For example, in (6), the topic *kwail-un* “fruit” and the NP in the comment *panana-lul* “banana” forms a superordinate-hyponymy relation. Once this semantic relation is violated the whole sentence would be unacceptable. For example, sentences in (7) and (8), Korean versions of (3) and (4), violate semantic constraints and are unacceptable. In (7), the topic panana-nun “banana” and the NP in the comment *kwail-ul* “fruit” form a hyponymy-superordinate relation; in (8), the topic *sakwa*-nun “apple” and the NP in the comment *panana-lul* form a sisterhood relation.

(7) *Panana-*nun* nay-*ka* kwail-*ul* kacang cohahanta.

Banana I fruit most love eat.

‘*Bananas, I like to eat fruit the most.’

(8) *Sakwa-*nun* nay-*ka* panana-*lul* kacang cohahanta.

Apple I banana most love eat.

‘*Apples, I like to eat banana the most.’

Despite these similarities, Korean has distinct characteristics from Chinese or English. One is that in Korean, the topic, the subject, and the object are all morphologically marked. When two NPs appear in the sentence-initial position, it is the morphological markers (–*un*/*nun*, -*i*/*ka*, -*ul*/*lul*) in Korean that decide the topic, the subject, or the object respectively. Chinese is different in that it lacks explicit morphological markers. 

Another difference is that Korean word order is much more flexible than English and Chinese. Korean flexible word orders are used for highlighting background information semantically or pragmatically [46]. In other words, the degree of freedom in word order depends on the style of speech. In formal speech, the word order is predominantly SOV, whereas in informal speech, a sentence can display one of the acceptable word orders depending on the intended pragmatic effects [47,48]. Therefore, a Korean sentence can be manipulated syntactically by changing morphological markers without changing the linear order of the sentences.

With these distinct characteristics, Korean has different language-specific parsing strategies. The role of Korean morphological marker is decisive in anticipating and processing Korean sentences [46,49,50]. As case-marking information is available early in verb-final sentences, it facilitates the parser to process sentences in predictive ways, not to wait until the verb is detected. In addition, according to Kim [50], monolingual Korean rely highly on morphology in processing sentences when being presented with multiple sources of cues, and Korean adults after 20 rely solely on morphology when interpreting sentences. Especially, the Korean topic marker, –*(n)un*, is believed to affect initial parsing and reparsing procedure [46].

In Chinese, both animacy and word order are important for syntactic processing [51,52]. In English, word order is the most basic device to indicate syntactic constituents. Liu et al. [53] showed that advanced L1-English L2-Chinese learners adopt English-based word order strategies in interpreting Chinese sentences; and English-Chinese bilinguals rely on a combination of word order and animacy in processing sentences.

As mentioned earlier, learners are influenced by L1-specific parsing strategies when processing L2 sentences. Given the distinct characteristics of Korean, the study makes a unique contribution to the field by focusing on the processing of Chinese BGT sentences by L1 Korean speakers, a learner group that has been under-researched in the literature. Different from the work of Yuan [25] which only included advanced L2 Chinese learners, the current study included both intermediate and advanced learners of Chinese, allowing us to further investigate the differences in processing Chinese BGT sentences between intermediate and advanced learners. In addition, the current study uses the eye-tracking technique, allowing us to capture and examine learners’ real-time processing of Chinese BGT sentences.

## 3. The Current Study

Investigating the processing of Chinese BGT sentences by intermediate and advanced L1-Korean L2-Chinese learners, the current study aims to attest to what extent the SSH is appropriate. According to the SSH [1,2], L2 learners tend to rely more on the lexical and semantic information but underuse syntactic information, presenting a “shallower” and different parsing pattern compared to native speakers. Specifically, unlike native speakers, L2 learners seldom adopt structure-based processing pattern when resolving ambiguities in L2 sentence processing. The current study attests these claims in two facets. The first facet regarding the syntactic processing focuses on how the topic and subject (the first NP and second NP) are processed, which belongs to the processing of temporary syntactic ambiguity. The SSH predicts that L2 learners would have different processing pattern compared to native speakers in solving syntactic ambiguity. The second facet concerning the semantic information explores whether Korean learners of Chinese are sensitive to the semantic constraint in processing the Chinese BGT sentences. If they can make good use of lexical semantic information in online sentence processing as predicted by the SSH, they would show sensitivity to the semantic constraints in processing Chinese BGT sentences like native speakers.

### 3.1. Participants

Participants included 62 L1-Korean speakers who were late learners of Chinese at a university in China and 37 native Chinese speakers. All the L2 learners had passed the Chinese proficiency test HSK 4, (HSK (Hanyu Shuiping Kaoshi) is a standardized language proficiency test of Mandarin Chinese for non-native speakers, administrated by Hanban, an agency of the Ministry of Education of the People’s Republic of China. In 2010, Hanban claimed that HSK 4 and HSK 6 correspond to the proficiency levels of B2 and C2 in Common European Framework of Reference for Languages (CEFR) respectively) and some of them had passed the HSK 6. They were also required to complete a background questionnaire and a Chinese proficiency test (The proficiency test consisted of multiple-choice questions, sentence making, and a cloze test with a total score of 40 points) before the experiment. Six Korean learners were excluded as they did not complete the whole proficiency test. Based on the test score, the remainder 56 learners were divided into intermediate and advanced group to better examine the possible different processing pattern between the two groups. Each group contains 28 learners. Their background information is presented in Table 1.

A one-way ANOVA revealed a significant difference in proficiency scores among the three groups (*F*(2, 90) = 15, *p* < 0.001). Post hoc Scheffé tests showed that the intermediate group scored significantly lower than the advanced group and the native group (*p* < 0.001), but no significant difference was found between the advanced learners and the native speakers (*p* = 0.154). Both the L2 learners and the native speakers had normal or corrected to normal vision.

### 3.2. Materials and Design

Following Yuan [25], four sentence types were involved in the experiment (see Table 2). Type A was a correct Chinese BGT sentence, with the topic (region 1) and its related NP (region 5) being in superordinate-hyponym relation. Type B and C were incorrect Chinese BGT sentences, because the topic (region 1) and its related NP (region 5) violate the semantic constraint forming a hyponym-superordinate relation and sisterhood relation respectively. Type D beginning with an adverb is not a BGT sentence, and it was grammatically and semantically acceptable serving as the baseline. Four types of sentences were divided into nine regions as in Yuan [25]. In the current study, each sentence type contained 32 sentences, with 128 target sentences altogether. All the target sentences were randomized together with 268 fillers in a Latin square design. The fillers were made up from correct Chinese adverbial clauses of time and cause, which were in the similar length to the target sentences. The Latin square design contained 4 lists, each of which had 99 sentences. Each list was assigned to 14 L2 learners and 9 native speakers, except for list 1 which was assigned to 10 native speakers. Each list was evenly taken up by the intermediate and advanced L2 learners.

Region 2 is considered as the first critical region with region 3 as its spillover region to examine how the topic and the subject (the first NP and the second NP) are analyzed by native Chinese speakers and Korean learners of Chinese. When processing region 2 in Types A, B, and C, participants were required to distinguish between a topic and a subject without the help of any morphological cues. In contrast, in region 2 of Type D, it may appear obvious that the NP in region 2, wo “I”, is a subject as an adverbial often precedes a NP or a subject in human languages [25]. As a result, region 2 in Types A, B and C is expected to require more effort to process than that of Type D.

Region 5 is the second critical region with region 6 as its spillover region. On the one hand, comparing region 5 in Type A and Type D aims to reveal whether there is syntactic reanalysis. Processing region 5 of Type D, does not involve any syntactic reanalysis due to the SVO structure. However, in processing region 5 of Type A, if the first NP is regarded as a base-generated topic, the NP in region 5 of Type D *xiangjiao* “banana” would be naturally considered as the object of the verb phrase, *aichi* “love to eat” without any syntactic reanalysis; if not, syntactic reanalysis would occur. On the other hand, exploring region 5 could examine whether participants were sensitive to the semantic constraint in processing Chinese BGT sentences. Being sensitive to the semantic constraint would mean that participants would have more difficulty in processing region 5 of Types B and C than that of Type A, as Type B and Type C violate the semantic constraint of Chinese BGT sentences. 

All the words used in the experiment were of high frequency and almost all of them were covered by the syllabus of Chinese Proficiency Test HSK 4. None of the participants reported difficulty in word recognition.

### 3.3. Apparatus

A computer program set up by Experiment Builder was used to present experimental sentences, which were displayed to participants on a 21-inch- wide Dell LCD monitor (resolution:1280 × 720 at 60 Hz) connected to a host computer. Each sentence was in black with Song 18-point font on a white background. Participants were seated approximately 65 cm away from the computer monitor. To minimize head movements during the experiment, participants were asked to stabilize their heads using the chin and forehead rest. Participants’ eye movements were recorded by an SR Eyelink 1000 plus system with the sampling rate of 1000 Hz. Eye movement data were collected from the right eye, while the participants read the sentences binocularly.

### 3.4. Procedure

During eye-tracking data collection, participants took part in the experiment one by one inside a quiet eye-tracking laboratory after the Chinese proficiency test. On arrival at the laboratory, participants were given detailed oral instructions in Chinese. Next, the eye-tracker was calibrated by a nine-point calibration, followed by a validation procedure. Each trial began with a fixation point located to the left of the first character of the sentence. Participants were required to first look at the fixation point. Once their fixation was confirmed by the eye-tracker, the test sentence would appear. They were asked to read the sentence silently and carefully. When they comprehended the sentence, they were required to press the space key, which would bring up a statement about the previous sentence on the screen. They were asked to judge whether the statement is true or false based on their understanding of the sentence by pressing “F” for false or “J” for true. To familiarize the participants with the procedures, they were provided with eight practice trials before starting the actual experiment. Experiments lasted on average 35 min. Eye movement data of all sentences were recorded, and the accuracy rates of their judgments were calculated.

### 3.5. Data Analysis

Following practices in previous studies [54,55,56], in the critical regions and spillover regions, fixations shorter than 80 ms and longer than 1200 ms were discarded. Besides, if an incorrect judgment was made after reading test sentences in Types A and D, the data of the previous test sentences were also excluded from the analysis. The excluded data accounted for 5%, 4% and 2.5% of all data for the intermediate learners, the advanced learners and the native Chinese speakers respectively. The remaining data were used for analysis.

The mean accuracy rates of judgment in Type A and Type D were 90% (SD = 2.1%) for the intermediate learners, 92% (SD = 3.2%) for the advanced learners, and 95% (SD = 1.8%) for the native Chinese speakers, implying that they were able to understand the test sentences in the experiment.

The eye-movement data were measured based on first fixation duration, first pass reading time, regression path duration, and total reading time in the current study. First fixation duration refers to the length of the first fixation made on a word or a region (all the reading measures in the current study were calculated based on specific regions as shown in Table 2 above). First pass reading time (also known as gaze duration) is the sum of all first-pass fixations on a region before eyes move out of the region to either the right or the left [57]. Both first fixation duration and first pass reading time are considered as indexes of lexical access, indicating how easily a word is recognized and retrieved from mental lexicon. It is also reported that first pass reading time can reflect predictability in the context, revealing the text integration process [57,58], and suggest semantic implausibility [36]. Regression path duration, also known as go-past time, refers to the total time spent fixating on the target itself and prior parts before participants’ eyes move past the target to the right, indicating syntactic integration and meaning integration [34,59]. Regression path duration is also an indicator of difficulty when first encountering the item and subsequent time taken to overcome that difficulty [60]. The total reading time means the total of all fixations made within a region of interest. It contains all the fixations which land on the target and imply the total time spent in reading the target [36]. It can be more influenced by contextual or discourse level factors [60].

### 3.6. Results

Within each participant group, a series of one-way ANOVA was first conducted to compare the four eye-tracking measures across Types. Post hoc Scheffé tests were used to further examine differences between Types.

#### 3.6.1. Native Chinese Speakers 

**Figure 1:**
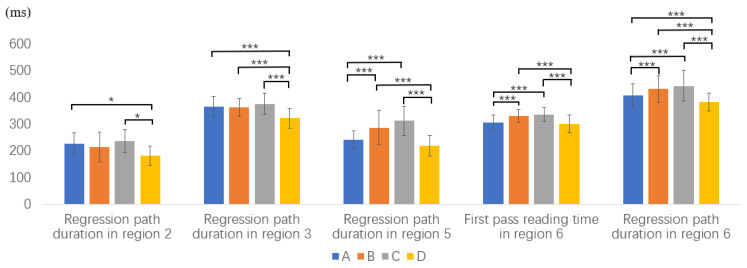
Part of representative reading measures for native Chinese speakers (* *p* < 0.05, *** *p* < 0.001).

The means and SDs of reading measures in the critical and spillover regions for the native Chinese speakers are presented in Table 3. Some of the representative significant results are shown in Figure 1.

In region 2, which is the first critical region taken by the subject NP, no significant overall difference was found among the four types in first fixation duration (*F*(3,140) = 0.25, *p* = 0.89), first pass reading time (*F*(3,140) = 1.13, *p* = 0.27), and total reading time (*F*(3,140) = 3.92 *p* = 0.1) from the native Chinese speakers. A significant overall difference was found in in regression path duration (*F*(3,140) = 5.87, *p* = 0.01). Post hoc Scheffé tests showed that regression path duration of Type D was significantly shorter than that of Type A and C.

To detect the possible spillover effects, we further explore region 3. Among the four measures, a significant overall difference was found in regression path duration among Types A, B, C, and D (*F*(3,140) = 11.1, *p* < 0.001). Post hoc Scheffé tests showed that regression path duration of Type D was significantly shorter than that in Types A, B and C. Longer regression path duration suggested that restructuring and reanalysis might have happened in in region 2 and spilled over to region 3 in Types A, B and C, but not in Type D.

In region 5, which is the second critical region taken by the object NP, a significant overall difference was found in first pass reading time (*F*(3,140) = 6.7, *p* < 0.001) and regression path duration (*F*(3,140) = 21.9, *p* < 0.001). Post hoc Scheffé tests showed that first pass reading time and regression path duration of Type B and C were significantly longer than those of Type D. Besides, regression path duration in Type A was significantly shorter than that in Type B and C.

We further analyze region 6 to examine the spillover effects. A significant overall difference was found in first pass reading time (*F*(3,140) = 12.1, *p* < 0.001), total reading time (*F*(3,140) = 12.9, *p* < 0.001), and regression path duration (*F*(3,140) = 34.2, *p* < 0.001). Post hoc Scheffé tests revealed that first pass reading time and total reading time in Type B and C were significantly longer than that in Type A and Type D. Post hoc Scheffé tests also showed that regression path duration in Type A was significantly shorter than that in Type B and C, and regression path duration in Type D was significantly shorter than that in Types A, B, and C.

Results from region 5 and region 6 indicate that the native Chinese speakers were sensitive to semantic constraints in processing region 5, and their syntactic reanalysis of region 5 spilled onto region 6. 

#### 3.6.2. Intermediate Learners of Chinese

**Figure 2:**
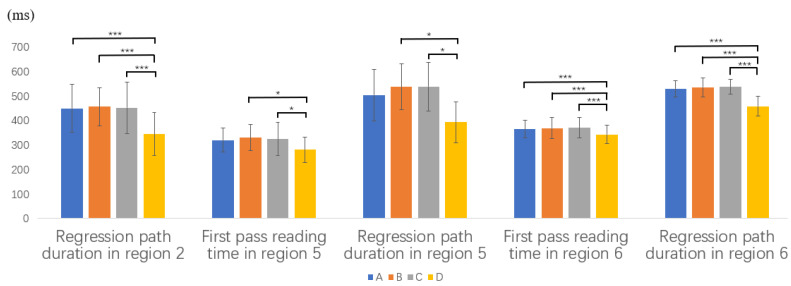
Part of representative reading measures for intermediate L2 learners (* *p* < 0.05, *** *p* < 0.001).

The means and SDs of reading measures in the critical and spillover regions for the intermediate learners of Chinese are presented in Table 4. Some of the representative significant results are shown in Figure 2.

In region 2, which is the first critical region taken by the subject NP, a significant overall difference was found in regression path duration (*F*(3,108) = 4.8 *p* = 0.003). Post hoc Scheffé tests revealed that regression path duration in Type D was significantly shorter than that in Types A, B, and C.

We further analyze region 4 to examine the spillover effects. A significant overall difference was found in regression path duration (*F*(3,108) = 34.72, *p* < 0.001). Post hoc Scheffé tests showed that regression path duration in Type D was significantly shorter than that in Types A, B, and C.

These findings indicated that similar to the native Chinese speaker group, reanalysis and restructuring took place in region 2, although the same word, wo ‘‘I’’ appeared across the four types. We suspected that the adverbials of time in region 1 in Type D made it easier for participants to analyze region 2 with little restructuring and reanalysis.

In region 5, which is the second critical region taken by the object NP, a significant overall difference was found in first pass reading time (*F*(3,108) = 4.5, *p* = 0.005) and regression path duration (*F*(3,108) = 4.8, *p* < 0.05). Post hoc Scheffé tests indicated that both first pass reading time and regression path duration in Type D were significantly shorter than that of Type B and C. 

We further analyzed region 6 to examine possible spillover effects. A significant overall difference was found in first pass reading time (*F*(3,108) =14, *p* < 0.001), regression path duration (*F*(3,108) = 31.56, *p* < 0.001) and total reading time (*F*(3,108) = 7.84, *p* < 0.001). Post hoc Scheffé tests showed that all of these three measures in Type D were significantly shorter than that in Types A, B, and C.

Based on the results from region 5 and region 6, it can be speculated that the intermediate learners’ syntactic reanalysis in region 5 of Types A, B, and C spilled over onto region 6, but their sensitivity to the semantic constraint of Chinese BGT sentences was not detected in either region 5 or region 6.

#### 3.6.3. The Advanced Chinese Learners

**Figure 3:**
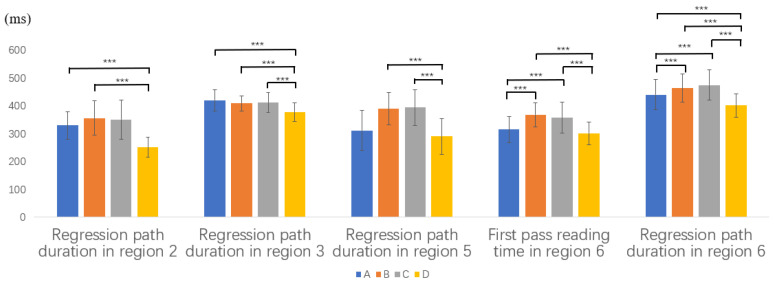
Part of representative reading measures for advanced L2 learners (*** *p* < 0.001).

The means and SDs of reading measures in the critical and spillover regions for the advanced Chinese learners are presented in Table 5. Some of the representative significant results are shown in Figure 3.

In region 2, which is the first critical region taken by the subject NP, a significant overall difference was found in first pass reading time (*F*(3,108) = 6.9, *p* < 0.001) and regression path duration (*F*(3,108) = 11, *p* < 0.001). Post hoc Scheffé tests showed that first pass reading time in Type D was significantly shorter than that in Type A and B. The same was true for regression path duration.

In region 3, the spillover region, we found a significant overall difference in regression path duration (*F*(3,108) = 8.5, *p* < 0.001). Post hoc Scheffé tests further showed that regression path duration in Type D was significantly shorter than that in Types A, B, and C. Similar to the native Chinese speakers and the intermediate learners, syntactic reanalysis was detected in their processing the second NP of Types A, B, and C.

In region 5, which is the second critical region taken by the object NP, significant overall differences were found in first pass reading time (*F*(3,108) = 5.4, *p* = 0.002) and regression path duration (*F*(3,108) = 18.7, *p* < 0.001). Post hoc Scheffé tests revealed that first pass reading time in Type C was significantly longer than that in Type A and D. The regression path duration of Type D was significantly shorter than that in Type B and C.

In region 6, the spillover region, significant overall differences were found in first pass reading time (*F*(3,108) = 12, *p* < 0.001) and regression path duration (*F*(3,108) = 11.5, *p* < 0.001). Post hoc Scheffé tests showed that first pass reading time in Type B and C was significantly longer than that in Type A and D. The regression path duration in Type D is significantly shorter than that in Types A, B, and C, and regression path duration in Type A was significantly shorter than that in Types B and C.

The above results indicate that the advanced learners have syntactic reanalysis in processing the second NP and were sensitive to the semantic constraint of Chinese BGT sentences. 

#### 3.6.4. Summary of the Findings 

In the first critical region, region 2 taken by the subject NP, the intermediate learners spent more time in processing in Types A, B, and C than Type D. The advanced learners and native Chinese speakers showed the similar patterns in region 3 but not region 2, indicating their syntactical reanalysis in region 2 spills over to region 3. Longer processing time on the second NP indicated that participants may tend to initially analyze the first NP as the subject, and syntactic reanalysis occurred when they confronted with the second NP.

In the second critical region, region 5 taken by the object NP, the native Chinese speakers spent more time on processing Type B and C than that in Type A, indicating their sensitivity to the semantic constraint of Chinese BGT sentences. The advanced learners showed similar processing patterns to native Chinese speakers in region 6, indicating that their sensitivity to semantic constraints in region 5 spills over to region 6. There was no indication that intermediate learners showed semantic sensitivity in either region 5 or region 6. In region 6, all three groups show a significantly longer processing time of Type A than that of Type D, implying that the syntactic reanalysis in region 5 spills over to region 6.

In sum, compared to native Chinese speakers, advanced Korean learners of Chinese, but not the intermediate learners, were sensitive to the semantic constraint of Chinese BGT sentences, although the intermediate learners demonstrated a native-like way of processing the topic and the subject of Chinese BGT sentences.

## 4. Discussion

According to the SSH, adult L2 learners fail to make full use of syntactic information and rely more on lexical-semantic and pragmatic information in processing. It claims that L2 learners’ sentence processing is qualitatively different from native speakers, since they seldom utilize structure-based processing strategies in solving the syntactic ambiguities in L2 processing. The results of the present study, however, suggest that Korean learners of Chinese utilize structure-based processing strategies in solving the syntactic ambiguities and they are sensitive to and can make use of syntactic information, but only advanced leaners are sensitive to semantic constraint in processing the Chinese BGT sentences.

### 4.1. Topic and Subject Identification

Known as a topic-prominent language with a variety of topic sentences, Chinese adopts the basic word order of SVO. In Chinese, both subjects and topics are common in sentence initial position, sharing many similarities although they are not the same. For instance, both topics and subjects in Chinese could be nouns without morphological markings. Therefore, the issue arises regarding how the parser would analyze the sentence-initial NPs. Li and Wu [61] conduct a self-paced reading task and report that native Chinese speakers tend to consider the sentence initial noun as a subject, but they are able to revise the subject-verb analysis to topic-subject analysis when indicative semantic or pragmatic information is sufficient.

For Korean learners of Chinese, without the cue of morphological markings, it may be challenging to distinguish topics from subjects. However, compared to the English-speaking L2 learners of Chinese with word-order-based processing strategy in Yuan [25], L1- Korean L2-Chinese learners with morphology-based processing strategy show similar performance in identifying the subject and topic while processing Chinese BGT sentences.

As shown in 3.7.2 and 3.7.3, a native-like syntactic reanalysis was found with Korean learners of Chinese when encountering the second NP in processing Chinese BGT sentences. Similar findings have been reported in Zeng et al. [33] on intermediate and advanced L1-Vietnamese L2-Chinese learners as well as in Yuan [25] on advanced L1-English L2-Chinese learners. It should be noted that English, unlike Chinese, doesn’t allow BGT sentences, and it’s rare to have two NPs in the sentence-initial positions. However, despite the topic-prominence of Chinese and Korean, both native Chinese speakers and L1-Korean L2-Chinese learners have similar performance in identifying between the topic and the subject. It is therefore postulated that the default analysis of the parser could be to process the sentence initial NP as the subject, whether a language is topic-prominent or not. Taken together with the native Chinese speakers’ similar processing pattern, it can be inferred that the syntactical reanalysis on the second NP can be explained by the application of the subject preference strategy [62]. This strategy refers to the processing system’s tendency to: (a) analyze ambiguous initial arguments as subjects, and (b) prefer subject-extractions over object-extractions in relative clauses. The current study is closely related to the former tendency. The subject preference strategy is considered as a universal parsing strategy as it has been well established in a number of languages such as Dutch [63], English [64], French [65], German [66,67], Italian [68,69], and Spanish [70]. Recently, it has also been reported for Turkish, an Altaic language [71]. Findings from this current study supports the first tendency claimed by this strategy as we argue that the nativelike processing pattern in analyzing the topic and subject found in the current study is facilitated by this universal parsing strategy. As a result, both intermediate and advanced L2 learners can be sensitive to the syntactic information in identifying the topic and subject, which is against the views of the SSH.

### 4.2. Sensitivity to the Semantic Constraint Underlying Chinese BGT Sentences

Overall, both the advanced and the intermediate learners had syntactic reanalysis in region 5 (or its spillover region, region 6) of Types A, B, and C, which is similar to the native speakers. This result indicates a demonstration of syntactic sensitivity by L2 learners. It is inferred that intermediate learners’ performance of syntactic reanalysis can be explained by the active filler strategy [72], a universal parsing strategy. This strategy describes that a parser, after identifying a potential filler, will actively search for a gap to assign the filler to. An active search process means that in processing subsequent words, the parser ranks the position of a gap above the option of a lexical noun phrase. Therefore, when a potential gap position appears, the parser will immediately associate it with the filler, which will lead to reanalysis if the next word reveals that there is no gap available in the possible position. In processing region 5 of Type A, after analyzing the first NP, *shuiguo* “fruit”, as a topic, the parser may actively search for a gap to assign the topic. After processing region 4, the parser might expect a potential gap rather than a noun. Therefore, when the parser was confronted with a noun, *xiangjiao* “banana”, instead of a gap, reanalysis occurred.

Regarding the intermediate learners’ insensitivity to the semantic constraint of the Chinese BGT sentences, we propose the following potential reasons. First, among different sources in processing, verbs have been regarded as the most important factor for anticipatory processing [73]. It is believed that the syntactic and thematic information associated with a verb plays a vital role in building the structure and meaning of a sentence [74]. In particular, the lexical meaning of a verb is an important clue to the prediction of the following constituent [75,76,77,78]. In the current study, the verb (*chi* “eat”)’s lexical meaning obviously predicts the following constituent to be something edible. Meanwhile, all the NPs in region 5 can be exactly collocated with the verb in region 4 (*chi* “eat”), which meets the prediction of the verb in region 4. It follows that processing difficulties arose for the intermediate learners to build up proper semantic constraint among the Types A, B, and C, where the NPs can all be collocated with the previous verb.

Second, it’s a characteristic of the human parser to match arguments with their appropriate predicates locally as quickly and readily as possible when building a semantic interpretation for the whole sentence [79]. The parser tends to attach new material to the phrase being processed rather than to a more distant position [80]. Here, regarding the less proficient intermediate L2 Chinese learners, it may be more difficult to relate region 5 to a more distant region, region 1, than to a local region, region 4, which would cost less processing effort. Due to their limited Chinese proficiency, it might be less challenging for the intermediate learners to associate the NPs in region 5 of Type B and C with the predicate chi “like to eat” than to associate it with the more distant NP in region 1.

Third, human memory is susceptible to interferences caused by similar constituents that must be recalled [81,82,83]. To establish the proper semantic constraint between region 1 and region 5 (For example *shuiguo* “fruit” and *xiangjiao* “banana” in Type A), the parser needs to recall the NP in region 1 when processing region 5. These two NPs are semantically similar concrete nouns, referring to things that can be eaten. Therefore, interferences and processing difficulties might have occurred in region 5 when the intermediate learners encountered information conveyed in the verb and must retrieve information from a highly correlated NP in region 1 to establish the correct semantic constraint. We argue that it might have been demanding for L2 learners to hold the first NP in memory for subsequent processing in order to integrate meaningful representation and to resist interferences at the same time. This is consistent with Cunnings [84]’s argument that L2 speakers are more susceptible to retrieval interference when successful comprehension requires access to information from memory.

The last reason accounting for intermediate learner’s insensitivity to semantic constraint could be that these learners might have difficulties in discarding the initial misinterpretation because they were not able to erase the memory trace of the initially established interpretation. According to Fujita and Cunnings [85], the lingering effects of the initial misinterpretation cannot be easily erased [85]. In the current study, the intermediate learners might have initially misinterpreted the NPs in Types B and C as the proper object of the previous verb, and subsequently have had difficulties in discarding the initial misinterpretation to rebuild proper semantic constraint with region 1. This lingering effect is also compatible with the good-enough processing model [86,87], which argues that readers do not always erase previously created representations that turn out to be incorrect.

## 5. Conclusions

To evaluate the SSH, the current study investigates the processing of a typical sentence structure in Chinese that contains no syntactic gap, the Chinese BGT sentence. Both the intermediate and advanced Korean learners showed syntactic reanalysis when identifying and differentiating between the subject and topic of Chinese BGT sentences, indicating their sensitivity to syntactic information in L2 sentence processing. The advanced, but not the intermediate learners demonstrated sensitivity to the semantic constraint in processing Chinese BGT sentences. We argue that the intermediate learners’ insensitivity to the semantic constraint may be related to the anticipatory role of the verb, and their limited ability to discard the initially assigned interpretation. The overall results provide evidence against the SSH. 

One limitation of the current study is that the L2 group may also be English learners, so they might not be complete L2 Chinese learners in a strict sense. To further test the SSH, the processing of Chinese topic sentences with and without a syntactic gap may be compared in future research. The relations between L2 learners’ grammatical knowledge and their on-line processing behavior remains to be further explored.

## Figures and Tables

**Table 1:** Background information of participants.

	Intermediate Learners Mean (SD)	Advanced Learners Mean (SD)	Native Speakers Mean (SD)
Proficiency test score (total = 40)	26.9 (4.3)	38.2 (2.1)	39.6 (2.6)
Age	21 (2.4)	22 (2.2)	31 (5)
Onset age of Chinese learning	18 (2.9)	18 (3.1)	N/A
Years of Chinese learning	4 (2.1)	5 (1.7)	N/A
Years of residence in China	3 (2.2)	5 (2.9)	N/A

**Table 2:** Sample set of experimental sentence types.

Types	Regions								
	1	2	3	4	5	6	7	8	9
A. BGT s-h	水果 fruit	我 I	最 most	爱吃 like eat	香蕉 banana	,所以 so	我 I	经常 often	买香蕉。 buy banana
B. BGT h-s	香蕉 banana	我 I	最 most	爱吃 like eat	水果 fruit	,所以 so	我 I	经常 often	买水果。 buy fruit
C. BGT s	苹果 apple	我 I	最 most	爱吃 like eat	香蕉 banana	,所以 so	我 I	经常 often	买香蕉。 buy banana
D. non BGT	以前 before	我 I	最 most	爱吃 like eat	香蕉 banana	,所以 so	我 I	经常 often	买香蕉。 buy banana

“s-h” stands for the superordinate-hyponym relation between the topic and its relevant NP in the comment. “h-s” stands for a hyponym-superordinate relation. “s” stands for sisterhood relation.

**Table 3:** Means (SD) of reading measures for the native Chinese speakers.

Region	1	First Critical Region 2	Spillover Region 3	4	Second Critical Region 5	Spillover Region 6
Sentence constituent	Topic NP (Adverb in Type D)	Subject NP			Object NP	
An example sentence in Type A	水果 fruit	我 I	最 most	爱吃 like eat	香蕉 banana	,所以 ,so
**First fixation duration (ms)**						
A. BGT s-h		142 (35)	235 (29)		165 (23)	237 (31)
B. BGT h-s		145 (36)	237 (27)		163 (25)	234 (40)
C. BGT s		144 (40)	239 (30)		161 (19)	251 (29)
D. non BGT		139 (44)	233 (28)		151 (24)	241 (34)
**First pass reading time (ms)**						
A. BGT s-h		191 (50)	295 (25)		206 (62)	306 (29)
B. BGT h-s		172 (32)	292 (31)		233 (51)	330 (23)
C. BGT s		183 (37)	301 (40)		235 (45)	335 (26)
D. non BGT		170 (47)	298 (32)		183 (47)	301 (33)
**Total reading time (ms)**						
A. BGT s-h		207 (34)	316 (30)		221 (32)	345 (30)
B. BGT h-s		199 (42)	311 (48)		242 (29)	370 (29)
C. BGT s		211 (40)	319 (55)		267 (30)	372 (38)
D. non BGT		175 (29)	303 (29)		209 (28)	343 (33)
**Regression path duration (ms)**						
A. BGT s-h		226 (40)	366 (38)		241 (34)	407 (43)
B. BGT h-s		213 (56)	363 (33)		287 (65)	431 (49)
C. BGT s		237 (43)	376 (40)		312 (54)	442 (57)
D. non BGT		181 (36)	322 (37)		219 (39)	383 (33)

**Table 4:** Means (SD) of reading measures for intermediate L2 learners.

Region	1	First Critical Region 2	Spillover Region 3	4	Second Critical Region 5	Spillover Region 6
Sentence constituent	Topic NP (Adverb in Type D)	Subject NP			Object NP	
An example sentence in Type A	水果 fruit	我 I	最 most	爱吃 like eat	香蕉 banana	,所以 ,so
**First fixation duration (ms)**						
A. BGT s-h		181 (29)	266 (45)		194 (31)	268 (34)
B. BGT h-s		191 (30)	260 (41)		218 (44)	259 (29)
C. BGT s		200 (45)	270 (44)		222 (29)	266 (23)
D. non BGT		188 (34)	273 (49)		216 (33)	267 (30)
**First pass reading time (ms)**						
A. BGT s-h		265 (45)	344 (45)		320 (50)	364 (36)
B. BGT h-s		269 (51)	351 (60)		330 (53)	368 (43)
C. BGT s		270 (41)	337 (34)		324 (67)	370 (42)
D. non BGT		237 (38)	336 (41)		280 (51)	343 (37)
**Total reading time (ms)**						
A. BGT s-h		331 (44)	396 (42)		411 (45)	429 (31)
B. BGT h-s		356 (33)	407 (44)		434 (56)	433 (36)
C. BGT s		340 (41)	392 (38)		478 (66)	435 (44)
D. non BGT		323 (29)	369 (41)		388 (40)	402 (31)
**Regression path duration (ms)**						
A. BGT s-h		449 (98)	486 (40)		503 (105)	529 (34)
B. BGT h-s		456 (77)	507 (39)		537 (94)	535 (38)
C. BGT s		452 (105)	479 (42)		538 (100)	537 (30)
D. non BGT		344 (88)	413 (19)		393 (84)	458 (40)

**Table 5:** Means (SD) of reading measures for advanced L2 learners.

Region	1	First Critical Region 2	Spillover Region 3	4	Second Critical Region 5	Spillover Region 6
Sentence constituent	Topic NP (Adverb in Type D)	Subject NP			Object NP	
An example sentence in Type A	水果 fruit	我 I	最 most	爱吃 like eat	香蕉 banana	,所以 ,so
**First fixation duration (ms)**						
A. BGT s-h		176 (34)	258 (34)		186 (21)	265 (34)
B. BGT h-s		180 (41)	257 (51)		200 (35)	255 (41)
C. BGT s		181 (35)	264 (39)		218 (24)	263 (39)
D. non BGT		156 (29)	265 (41)		190 (21)	261 (44)
**First pass reading time (ms)**						
A. BGT s-h		220 (46)	306 (43)		242 (54)	314 (47)
B. BGT h-s		225 (30)	295 (30)		282 (53)	368 (43)
C. BGT s		198 (33)	308 (31)		286 (43)	357 (56)
D. non BGT		184 (33)	294 (28)		241 (70)	301 (41)
**Total reading time (ms)**						
A. BGT s-h		254 (54)	346 (31)		287 (33)	391 (44)
B. BGT h-s		301 (48)	342 (29)		322 (37)	408 (52)
C. BGT s		288 (35)	344 (41)		341 (43)	410 (59)
D. non BGT		240 (41)	333 (39)		286 (32)	378 (40)
**Regression path duration (ms)**						
A. BGT s-h		329 (50)	419 (38)		311 (72)	439 (54)
B. BGT h-s		355 (62)	408 (27)		389 (59)	463 (51)
C. BGT s		350 (70)	411 (35)		393 (64)	474 (55)
D. non BGT		251 (35)	377 (33)		290 (64)	401 (42)

## Data Availability

The data for the current research are stored in the Eye-tracking Laboratory at School of Foreign Languages, Shanghai Jiao Tong University and are accessible upon reasonable request.
